# Structure of the ribosome post-recycling complex probed by chemical cross-linking and mass spectrometry

**DOI:** 10.1038/ncomms13248

**Published:** 2016-11-08

**Authors:** Kristin Kiosze-Becker, Alessandro Ori, Milan Gerovac, André Heuer, Elina Nürenberg-Goloub, Umar Jan Rashid, Thomas Becker, Roland Beckmann, Martin Beck, Robert Tampé

**Affiliations:** 1 Institute of Biochemistry, Biocenter, Goethe University Frankfurt, Max-von-Laue-Str. 9, 60438 Frankfurt a.M., Germany; 2 Structural and Computational Biology Unit, EMBL Heidelberg, Meyerhofstr. 1, 69117 Heidelberg, Germany; 3 Gene Center and Center for Integrated Protein Science Munich (CiPSM), Department of Biochemistry, University of Munich, Feodor-Lynen-Str. 25, 81377 Munich, Germany

## Abstract

Ribosome recycling orchestrated by the ATP binding cassette (ABC) protein ABCE1 can be considered as the final—or the first—step within the cyclic process of protein synthesis, connecting translation termination and mRNA surveillance with re-initiation. An ATP-dependent tweezer-like motion of the nucleotide-binding domains in ABCE1 transfers mechanical energy to the ribosome and tears the ribosome subunits apart. The post-recycling complex (PRC) then re-initiates mRNA translation. Here, we probed the so far unknown architecture of the 1-MDa PRC (40S/30S·ABCE1) by chemical cross-linking and mass spectrometry (XL-MS). Our study reveals ABCE1 bound to the translational factor-binding (GTPase) site with multiple cross-link contacts of the helix–loop–helix motif to the S24e ribosomal protein. Cross-linking of the FeS cluster domain to the ribosomal protein S12 substantiates an extreme lever-arm movement of the FeS cluster domain during ribosome recycling. We were thus able to reconstitute and structurally analyse a key complex in the translational cycle, resembling the link between translation initiation and ribosome recycling.

## Abstract (web summary)

Ribosome recycling orchestrated by ABCE1 connects translation termination and mRNA surveillance mechanisms with re-initiation. Using a cross-linking and mass spectrometry approach, Kiosze-Becker *et al*. provide new information on the large conformational rearrangements that occur during ribosome recycling.

## Graphical abstract

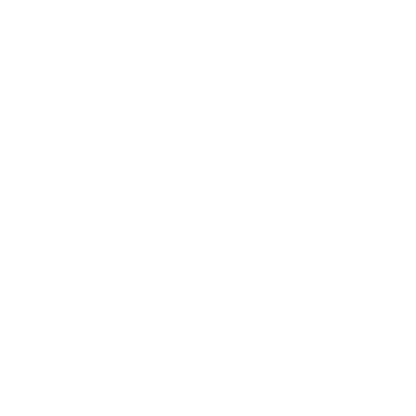

Ribosome-driven protein biosynthesis is a cyclic process, which comprises four steps: initiation, elongation, termination and recycling^1^,^2^,^3^. In Eukarya and Archaea, the ATP binding cassette (ABC) protein ABCE1 catalyses the essential step of ribosome recycling by splitting the ribosome into its small 40/30S and large 60/50S subunits^4^,^5^,^6^. Hence, ABCE1 emerges as the missing link between termination and initiation by potentially coordinating the re-initiation via the released 40/30S·ABCE1 complex, named post-recycling complex (PRC), where ABCE1 remains bound after ribosome splitting until ATP hydrolysis has occurred^2^,^4^,^7^. Structural insights of ABCE1 have recently become available, for example, by X-ray structures of ABCE1 as well as cryo-electron microscopy (cryo-EM) analyses of termination/pre-recycling complexes^4^,^8^,^9^,^10^. However, the structure of the PRC and conformational changes during ribosome recycling remain elusive up to the present day.

ABCE1 is one of the most conserved proteins and it is essential for life in all Eukarya and Archaea examined so far^11^,^12^,^13^. It is the sole member of the subfamily E within the superfamily of ABC proteins^14^. ABCE1 is equipped with two nucleotide-binding domains (NBDs) oriented in a head-to-tail fashion and connected via hinge 1 and 2 region^4^,^9^. Furthermore, it contains a unique N-terminal FeS cluster domain, aligned by two diamagnetic [4Fe–4S]^2+^ clusters^15^. ABCE1 was originally classified as RNase L inhibitor 1 (RLI1) in antiviral ribonucleic acid (RNA) immunity and as host protein 68 (HP68) required for HIV capsid assembly in human cells^16^,^17^. Nevertheless, in accordance to its strong sequence conservation, ABCE1 proved to be indispensable for the fundamental process of ribosome recycling^2^,^5^. ABCE1 is able to recycle post-termination complexes after canonical translation as well as vacant ribosomes and stalled ribosomal complexes, which are further processed by messenger RNA (mRNA) surveillance mechanisms^2^,^18^,^19^,^20^,^21^. During canonical translation, ABCE1 is recruited to the post-termination complex after dissociation of the GTPase eRF3/aEF1α (ref. 8). It is anticipated that ABCE1 goes through a tweezer-like motion typical of ABC proteins, cycling between stages of closing and opening of the NBD interface triggered by ATP binding and hydrolysis, respectively^22^,^23^. On ATP binding, the closing of the NBDs presumably forces the FeS cluster domain to swing out of the NBD cleft into the inter-subunit space of the ribosome, which tears the ribosomal subunits apart either directly or via the bound eRF1/aRF1 or e/aPelota^8^. Hence, the released subunits are now available for a new translation round^24^. Notably, ABCE1 itself remains bound within the PRC (40S/30S·ABCE1·ATP) until ATP is hydrolysed, and might assist here in the re-initiation via the reported interactions with initiation factors^4^,^12^,^25^.

Up to now, only pre-recycling complexes have structurally been resolved by cryo-EM, demonstrating that ABCE1 binds to the translational GTPase binding site and adopts a semi-closed conformation^8^,^10^,^26^. The overall conformation of ABCE1 within the canonical termination/pre-recycling complex (80S·eRF1·ABCE1) as well as in the pre-recycling state within mRNA surveillance (80S·ePelota·ABCE1) is very similar^8^,^10^,^26^. In both cases, ABCE1 establishes various contacts to the small ribosomal subunit and minor contacts to the large ribosomal subunit^8^,^10^. Still, the location of ABCE1 and conformational changes in all sequent steps along the recycling process, especially the post-splitting state as platform for re-initiation, remains elusive so far. Termination and ribosome recycling are multi-step processes consisting of several sub-steps including the 80S/70S termination complex, with the pre- and post-peptidyl-hydrolysis state accompanied by peptide release, the post-termination/pre-recycling step followed by the PRC (addressed here), which further includes steps such as ribosome splitting, e/aRF1 release and recycling of mRNA and transfer RNA (tRNA). Furthermore, the exact role and movement of the FeS cluster domain during ribosome recycling are not understood yet. Attempts to determine the structure of 40S/30S·ABCE1·ATP complexes have failed, likely due to the complexity and variability of the 40S/30S subunit as well as to the short-lived nature of this intermediate state.

XL-MS studies provide an advanced technique to discover the site of protein interactions as well as transient binding partners and to construct protein interaction networks. This approach has been recently applied to reveal the architecture of the nuclear pore complex, the 26S proteasome, the protein phosphatase 2A network, polymerase II complexes and various others^27^,^28^,^29^,^30^. Moreover, it contributed in a hybrid approach of low-resolution structural methods to the dissection of the molecular architecture of the 40S·eIF1·eIF3 translation initiation complex, characterized by a number of transient RNA–protein interactions^31^. Stable and rigid core complexes are often resolved by crystallography, whereas the positions of additional, peripheral factors, such as ABCE1 on the ribosome, are mapped by cross-linking approaches or cryo-EM^28^.

Here, we combined chemical cross-linking with mass spectrometry (XL-MS)^32^ to address the architecture of the PRC (30S·ABCE1). In addition, we reconstructed the PRC at low resolution by cryo-EM. Using a homogeneously purified population of the 1-MDa PRC composed of 16S ribosomal RNA (rRNA), 28 ribosomal (r-)proteins and ABCE1 stably arrested by non-hydrolysable AMP-PNP, we mapped the position of ABCE1 within this multisubunit ribonucleoprotein particle by means of XL-MS. AMP-PNP is crucial for the preparation of a post-splitting complex as (i) ATP hydrolysis triggers the release of ABCE1 from the small subunit and (ii) ADP is unable to induce conformational changes of ABCE1 required for ribosome binding^4^,^5^. Notwithstanding, taking a two-step mechanism with two distinct nucleotide-binding events into account, AMP-PNP prevents the second step, the splitting process, because ABCE1 is trapped in the first termination step and cannot proceed to the splitting step^6^. Hence, the PRC can be experimentally addressed only by the reverse reaction by AMP-PNP dependent occupation of small ribosomal subunit by ABCE1. Further, ABCE1 is able to split translationally inactive ribosomes, for example, vacant or starved (Stm1 occupied) ribosomes^20^,^21^. Hence, mRNA or tRNA, which is released during ribosome splitting, are not essential for the PRC studied in the present context^5^.

Following the two independent structural approaches, namely XL-MS and cryo-EM, we demonstrate that ABCE1 remains bound at the translational GTPase binding site after ribosome splitting, contacting the S24e protein of the small subunit. Notably, the FeS cluster domain of ABCE1 undergoes a large rotational and translational rearrangement towards the ribosomal protein S12 on nucleotide-dependent closure of the NBDs. Thus, we were able to dissect a key complex in the mRNA translation process, which turns into a cyclic process by connecting translation initiation to termination/recycling events.

## Results

### Preparation of the post-recycling complex

**Figure 1:**
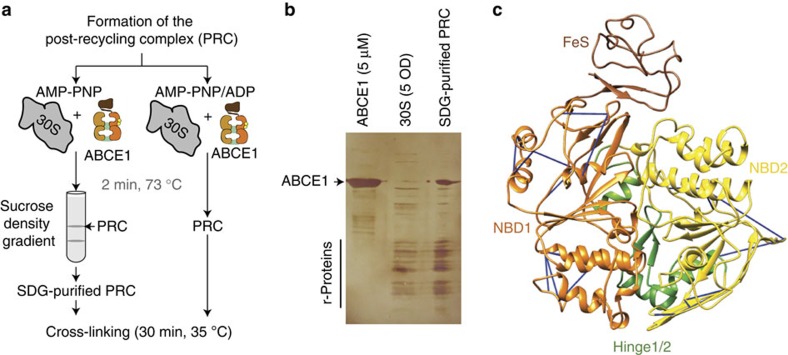
Lysine-specific cross-linking of ABCE1 bound in the post-recycling complex (PRC). (**a**) A stably arrested and homogeneous population of PRC was isolated from sucrose density gradients (SDG) after reconstitution from purified components at physiological temperatures and in the presence of non-hydrolysable AMP-PNP. (**b**) Sample quality was analysed via SDS–polyacrylamide gel electrophoresis (silver-stain). Alternatively, PRCs were reconstituted under identical conditions from isolated components without any additional purification via SDG. As control, the sample was prepared in the presence of ADP, which does not promote a stable arrest of ABCE1 on the small ribosomal subunit. (**c**) Lysine specific cross-linking with DSS resulted in a distinct set of intra cross-links within ABCE1. Cross-links shown here are those of the SDG-purified samples (closed model).

The structure of the post-recycling/post-splitting complex is of crucial importance in understanding the recycling process and the subsequent re-initiation of mRNA translation. As the cryo-EM and X-ray analyses of the post-splitting complex remained notoriously difficult, we probed the architecture of the PRC by chemical cross-linking in combination with mass spectrometry (XL-MS). An essential prerequisite in the structural analysis of the PRC is a stably arrested, homogeneous population of ABCE1 trapped at the small ribosomal subunit. We established this using the non-hydrolysable ATP analogue AMP-PNP in combination with sucrose density gradient (SDG) centrifugation to arrest ABCE1 in the closed state on the small ribosomal subunit and to separate the 30S·ABCE1·AMP-PNP complex from non-assembled components, respectively (Fig. 1a,b, Supplementary Fig. 1a). Alternatively, we assembled the post-splitting complex under identical conditions without SDG centrifugation. This approach allowed us to directly compare the assembly of the PRC in the presence of AMP-PNP or ADP, the latter of which does not promote ribosome recycling and prevents a stable arrest of ABCE1 on the small ribosomal subunit^4^. Assembled complexes were subsequently cross-linked under identical conditions using either a 30- or 80-fold molar excess of the isotope-coded amine-specific cross-linker disuccinimidyl suberate (2 mM or 5 mM DSS, d0/d12). The monodispersity and homogeneity of each sample were checked by immunoblotting and negative-stain EM, respectively (Supplementary Fig. 1). Subsequent proteolysis resulted in a complex mixture of tryptic peptides, which were analysed by tandem mass spectrometry and identified using the xQuest/xProphet tool searching against a database containing the protein sequences of ABCE1 and all 28 proteins of the small ribosomal subunit from *Sulfolobus solfataricus* (Supplementary Data; Supplementary Table 1)^30^,^33^.

### XL-MS analysis of the post-recycling complex

**Figure 2:**
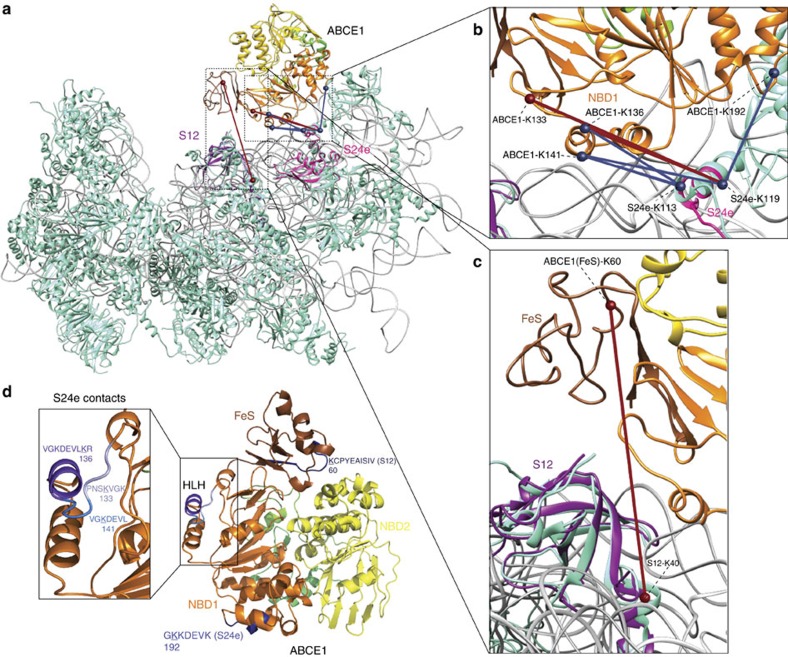
Architecture of the PRC (30S·ABCE1·AMP-PNP) mapped by XL-MS. (**a**) The orientation of ABCE1 in the PRC based on the identified inter cross-links with the archaeal ribosomal proteins S24e (pink, **b**) and S12 (dark magenta, **c**), depicted in blue and red lines. Blue lines indicate cross-links with a length<30 Å and red lines cross-links>30 Å. Identified inter cross-links were certified using an *in silico* model of the *S. solfataricus* 30S constructed by aligning the homology models of the archaeal ribosomal proteins to the small ribosomal subunit from *S. cerevisiae* (pdb: 3U5G/F, r-proteins: cyan, rRNA: grey) and positioning ABCE1 according to the cryo-EM map of the rescue/pre-recycling complex (pdb: 3J16). (**d**) The major contact area of ABCE1 towards the 30S primarily locates in the helix–loop–helix region (HLH).

Using the XL-MS approach, we analysed the arrested PRC and successfully identified 56 inter-protein cross-links across all samples analysed. Thereof, 22 are cross-links between ABCE1 and ribosomal proteins, and all the remaining cross-links are found between r-proteins (Table 1; Supplementary Table 2, Supplementary Fig. 2). The number of identified cross-links is in line with recent analyses of ribonucleoprotein complexes^34^. A detailed analysis of the SDG-purified PRC (30S·ABCE1·AMP-PNP) cross-linked with 2 mM or 5 mM DSS (30- or 80-fold molar excess of cross-linker) revealed 63 intra-ABCE1 cross-links (Fig. 1c), and more important 33 inter-protein cross-links, resulting in eight unique Cα–Cα restraints between r-proteins and eight distinct restraints between ABCE1 and r-proteins (Fig. 2). Additionally, we were able to identify in all samples 138 intra-ABCE1 cross-links as well as a quantity of 28 mono-links to lysine residues of ABCE1 (Supplementary Table 3, Supplementary Fig. 3). These results derived from the combination of two independent preparations of the PRC with multiple samples per preparation. Independently of their purification approach, all three SDG-purified samples as well as the two samples prepared in presence of AMP-PNP resulted in the same major cross-links between ABCE1 and S24e. The statistics of identification of intra cross-links within ABCE1 (Supplementary Table 3) and all the inter-protein cross-links are provided (Supplementary Table 2).

Inter and intra cross-links were further validated by analysing the distances between the two cross-linked lysine residues on a homology model of the 30S subunit from *S. solfataricus*. Thus, homology models of each known ribosomal protein from *S. solfataricus* (Supplementary Table 1) were constructed using Phyre^2^ (Protein Homology/Analogy Recognition Engine V 2.0)^35^. To construct the 30S of *S. solfataricus in silico*, the homology models of the archaeal ribosomal proteins were aligned to the known small ribosomal subunit from *Saccharomyces cerevisiae* (pdb: 3U5G, 3U5F)^36^. Obtained cross-links were then analysed and certified using the XlinkAnalyzer tool for Chimera^37^. Yeast ribosomal proteins are thereby named according to the new nomenclature of ribosomal proteins, while the archaeal r-proteins hold their UniProt entry name going along with the MS analysis^38^. ABCE1 itself is positioned according to the cryo-EM map of the pre-recycling complex (pdb: 3J16)^8^. The median Cα–Cα distance for all obtained cross-links is 17 Å, with 83.9% of the distances below 30 Å, respectively (Supplementary Fig. 2a). When only cross-links between ribosomal proteins are considered, 32 out of the 34 identified inter-protein cross-links (94.1%) displayed a Cα–Cα distance between cross-linked lysines<30 Å. The estimated average for the DSS cross-linker lies at 17 Å with a maximum threshold at 30 Å, accounting for cross-linked side-chains, protein flexibilities and model inaccuracies^29^,^32^,^39^. Thus, we are able to demonstrate reliable and reproducible inter-protein cross-links between ABCE1 and especially the S24e r-protein. Further, the identified inter-protein ribosomal cross-links connect structurally adjacent ribosomal proteins, confirming the reliability of the acquired results (Supplementary Fig. 2b). Inter cross-links exceeding the expected distance mainly occur in samples that were not separated via SDG and that likely contained a conformationally heterogeneous population of PRCs (Supplementary Table 2). The two cross-links exceeding the 30 Å maximum thresholds, as for example the 63.9 Å cross-link between the N-terminal region of the ribosomal protein S30e (position 9) and the central region of the S5 protein, can be explained by poor homology models (performed by Phyre^2^). The structure of the archaeal S30e is not well defined. In particular, the N- and C-terminal regions of the ribosomal proteins, which are cross-linked, are often less conserved between species and, thus, affect accuracy of the homology models. This explains the uncertainty in the length of the cross-link. The same argument holds true for the 33.8 Å crosslink between S3A and the carboxy terminus of S28.

The obtained intra cross-links of ABCE1 were analysed using an available crystal structure and a model of the closed state, revealing an even distribution, surface accessibility and valid distance constraints (Fig. 1c, Supplementary Fig. 3)^4^,^9^. Noteworthy, we do not see any intra cross-links between both NBDs, spanning the NBD cleft. Notably, a majority of the seemingly violated intra-ABCE1 cross-links (red, ≥25 Å) originated from cross-links to the FeS cluster domain (Supplementary Fig. 3a), supporting the notion that this domain is highly dynamic^8^,^9^. The set of obtained mono-links confirms the solvent accessibility of the ABCE1 surface and the reactivity of the lysines with respect to the cross-linker. All mono-links are thereby evenly distributed over the protein surface, limiting solid conclusions about the interaction sites with the post-splitting complex via a protected region (Supplementary Fig. 3b). To conclude, using the XL-MS approach, we obtained a significant set of inter-protein cross-links between ABCE1 and r-proteins, which allows us to dissect the ABCE1-binding site in the PRC.

### Structural organization of the post-recycling complex

We mapped the position of ABCE1 on the PRC by XL-MS and identified eight prominent cross-links of ABCE1 to the archaeal S24e and S12 ribosomal proteins (Fig. 2a–c, Supplementary Table 2). Lysines 133, 136, 141, 153 and 192 of ABCE1, most of them residing in the helix–loop–helix (HLH) region (aa 132-161; Fig. 2d), form cross-links with lysine 113 or 119 of the ribosomal subunit S24e (Table 1). In addition, lysine 60 of the FeS cluster domain (ABCE1) cross-links with lysine 40 of the ribosomal protein S12 (Fig. 2c). Thus, the identified ABCE1-binding site at the small ribosomal subunit is confined to two proteins (S24e and S12), which are highly conserved in Archaea, yeast and humans (eS24 and uS12 according to the new nomenclature)^38^. The S24 cross-links were confirmed by two independent preparations of the PRC with a number of different samples per preparation, with two unique restraints consistent across independent replicates. Importantly, two of these most prominent restraints to the S24e r-protein were consistently identified using different cross-linker amounts and complexes prepared in the presence of AMP-PNP without separation by SDG (Supplementary Fig. 4). Moreover, reliable cross-links were not detected when ABCE1 and 30S were analysed in the presence of ADP (Supplementary Table 2).

**Figure 3:**
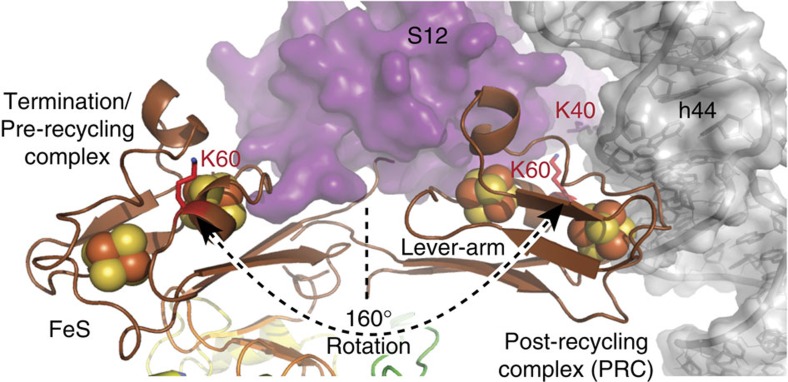
Extensive movement of the FeS cluster domain. The FeS cluster domain, anchored to NBD1 via a two β-strand lever arm, swings out of the NBD cleft and converges towards the 30S subunit to occupy a cleft between the S12 r-protein and rRNA (h44) of the small ribosomal subunit. Due to this conformation change, the Cα–Cα distance between these highly conserved lysines in Archaea, yeast and human is reduced from 59.5 Å in the pre-splitting state to 17.5 Å in the post-recycling state.

Valid distances of all cross-links to S24e (11–40 Å) were confirmed using our model of *S. solfataricus* 30S. In particular, the unique HLH region of ABCE1 plays here a major role within the formation of the PRC (Fig. 2b, d). Furthermore, the cross-link between S12 and ABCE1 was identified in two independent samples (30- or 80-fold molar excess of DSS) of one preparation and within four technical replicates (two per condition; Supplementary Fig. 5). Considering that the predicted cross-link distance in the pre-splitting complex should be 59.5 Å (Fig. 2c), this post-splitting contact could be established by a large conformational movement of the FeS cluster domain, resulting in a repositioning of the FeS cluster domain closer to the A site where ribosomal subunit S12 is located (Fig. 3). It is worth mentioning that the FeS cluster domain is very small (75 aa) and harbours only seven lysines. Since five of them locate on the opposite site of the FeS cluster domain compared with lysine 60 and cross-linking of the neighbouring lysine 59 prevents trypsin cleavage, the cross-link from ABCE1 (lysine 60, fragment KCPYEAISIVNLPDELEGEVIHR) to the S12 ribosomal subunit (lysine 40, fragment EKYDPLGGAPMAR) reproducibly found in four technical replications is of high significance (Supplementary Table 2, Supplementary Fig. 5).

**Figure 4:**
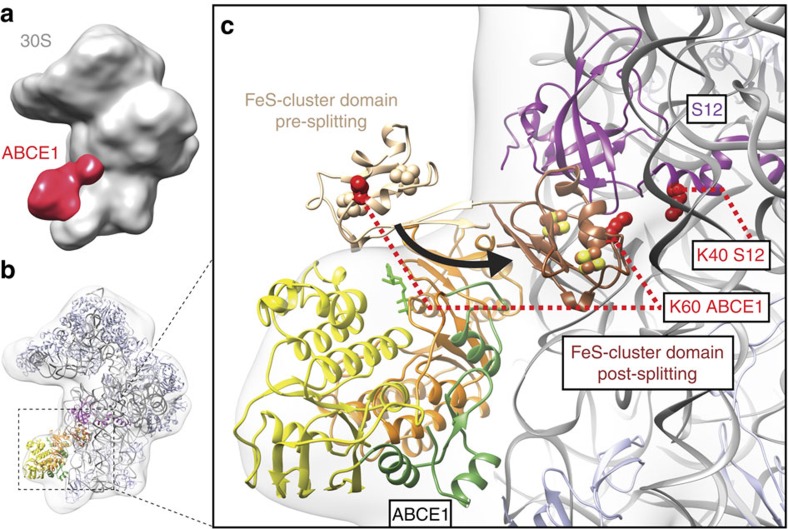
Low-resolution cryo-EM structure of the 30S·ABCE1 post-splitting complex. (**a**) Overview of the 30S·ABCE1 post-splitting complex electron density map low-pass filtered at ∼25 Å. The final 30S·ABCE1 data set contained 19,500 particles and the final resolution was 17 Å (Fourier shell correlation 0.5). The ABCE1 extra density is shown in red. (**b**) Model of the 30S·ABCE1 complex in post-splitting state showing the models of the *P. furiosus* small 30S subunit (grey; 4V6U)^52^ and ribosome-bound ABCE1 (FeS cluster domain brown; NBD1 orange and NBD2 yellow; hinges 1 and 2 green, ADP-bound green; 3J15)^8^. The FeS cluster domain was fitted into the extra density located near ribosomal proteins S12 (purple). (**c**) Zoom-in showing the pre-splitting (wheat) and post-splitting (brown) state of the FeS cluster domain. The post-splitting state was modelled based on a specific inter-crosslink in XL-MS between lysine 60 of ABCE1 (lysine 64 in *P. furiosus*) and lysine 40 of S12 (shown in red). Because of this conformation change, the Cα–Cα distance between these highly conserved lysines in Archaea, yeast and human is reduced from 59.5 Å in the pre-splitting state to 17.5 Å in the post-splitting state.

To provide a second, independent line of evidence for the position of ABCE1 and the extreme structural reorganization of the FeS cluster domain in the PRC, we analysed the archaeal 30S·ABCE1·AMP-PNP complex by cryo-EM. In spite of the facts that archaeal 30S ribosomal particles were up to now not accessible to cryo-EM analyses and occupancy was low, we resolved the structural architecture of the PRC by a low-resolution cryo-EM reconstruction, in which, indeed, an extra density near rRNA helix 44 (h44) and S12 was observed (Fig. 4). The small subunit is well-known for orientation bias and inhomogeneity by dimerization and aggregation in negative stain. While the two NBDs fit into the body part of the ABCE1 density as shown in the pre-splitting state, confirming the cross-links between the HLH region and the ribosomal subunit S24e, there was no visible density for the FeS cluster domain in the pre-splitting position. Notably, with a 160-degree rotation of the FeS cluster domain from the pivot point (proline 76), the extra density near S12 and h44 could be easily positioned in a way that explains the cross-link data described above (Fig. 4). The orientation of the FeS cluster domain is based on positioning lysine 60 of ABCE1 and lysine 40 of S12 at a Cα–Cα distance of 17.5 Å, using cross-linker and lever length as restraints. Because of this conformation change, the Cα–Cα distance between these highly conserved lysines in Archaea, yeast and human is reduced from 59.5 Å in the pre-splitting state to 17.5 Å in the post-recycling state. Thus, the low-resolution cryo-EM structure of the archaeal PRC undoubtedly corroborates the conformational reorganization of ABCE1 in the PRC complex as revealed by XL-MS.

A closer inspection of all identified cross-links from ABCE1 reveals that almost all contacts to the small ribosomal subunit are established via NBD1 and the FeS cluster domain. Based on this ribosome splitting-persistent contact between the HLH motif of NBD1 in ABCE1 and the ribosomal subunit S24e (eS24 in yeast), the cross-link between the FeS cluster domain and the ribosomal subunit S12 (uS12 in yeast) becomes highly relevant in explaining the large conformational rearrangement of the FeS cluster domain during ribosome recycling.

## Discussion

**Figure 5:**
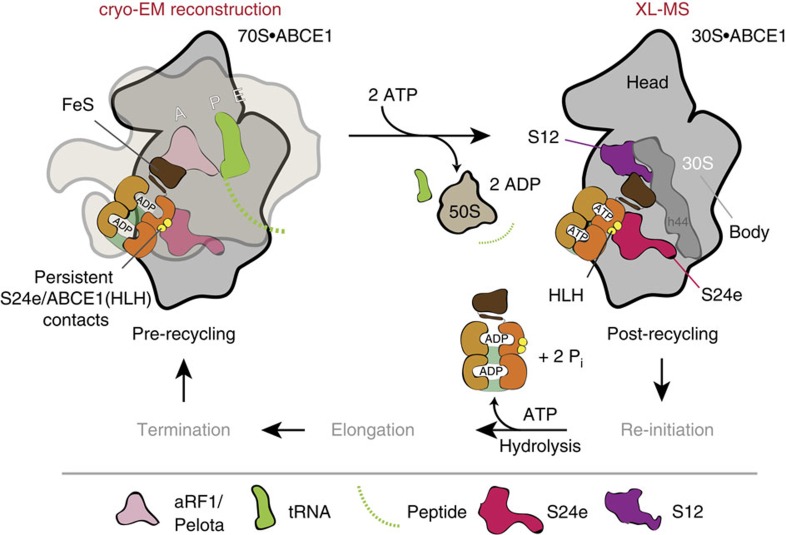
Conformational changes of ABCE1 during ribosome recycling. During the cyclic process of translation, post-termination/pre-recycling complexes occur, which need to be recycled into their components to be available for the subsequent re-initiation. After e/aRF3 dissociation, ABCE1 binds to the GTPase binding site of these complexes, establishing contacts to the r-proteins of the large and small subunit (P0, L9, S24, S6)^8^. ATP occlusion of ABCE1 leads to major conformational changes, especially a large rotational and translational repositioning of the FeS cluster domain, which splits the ribosomal subunits apart—either directly or via the bound e/aRF1. ABCE1 itself remains bound to the small subunit until ATP is hydrolysed (PRC). Consequently, the contacts to proteins of the large subunit are released and major contacts to the proteins of the small subunit like S24e are preserved. Additionally, a new contact to the S12 protein is established, caused by the large rotational and translational movement of the FeS cluster domain, anchoring ABCE1 on the 30S.

In this study, we reconstituted and structurally dissected the PRC (30S·ABCE1·AMP-PNP) using a combined cross-linking and mass spectrometry approach. We provide direct evidence that ABCE1 establishes major contacts with the S24e ribosomal protein in the PRC, demonstrating that the recycling factor remains bound at the so-called translational GTPase binding site after ribosome splitting. Thus, the connectivity map (Fig. 2) largely recapitulates recent cryo-EM structures of the yeast and mammalian pre-recycling complex, which pointed out a related binding site of ABCE1 at the GTPase center contacting ribosomal proteins S24e and S6e as well as rRNA (h5, h8, h14 and h15) on the small ribosomal subunit^8^,^10^,^26^. These findings imply that ABCE1, despite unaltered ribosomal contact sites of NBD1 before and after splitting, undergoes large conformational changes during ribosome splitting. Based on the unexpected finding of the statistically significant cross-link between the FeS cluster domain of ABCE1 (lysine 60) and the S12 (lysine 40) ribosomal protein, we infer a 160-degree rotation. This extensive rearrangement of the FeS cluster domain brings lysine 60 of ABCE1 in cross-linking distance to lysine 40 of the S12 subunit (Fig. 3). The cross-link of the FeS cluster domain to the S12 r-protein is in perfect agreement with our low-resolution cryo-EM data (Fig. 4). We therefore anticipate that ABCE1 undergoes a tweezer-like movement as other ABC proteins. On NBD closure, the FeS cluster domain, anchored to NBD1 via a two β-strand lever-arm, swings out of the NBD cleft and converges towards the 30S subunit to occupy a cleft between the S12 r-protein and rRNA (h44) of the small ribosomal subunit (Fig. 4). The FeS cluster domain remains anchored in the groove between S12 and rRNA (h44) until ATP is hydrolysed by one or both NBDs, which releases the tensed lever-arm and allows the FeS cluster domain to swing back into its resting position, illustrated by the X-ray structure of the open state of ABCE1 (ref. 9). So, ABCE1 can dissociate from the small ribosomal subunit primed for a subsequent round of translation (Fig. 5).

The fact that NBD1 remains bound to the small subunit after ribosome splitting enables ABCE1 to act as a platform for subsequent re-initiation via its known interactions with initiation factors^12^. By occupying the ribosomal subunit interface, ABCE1 may prevent ribosomal subunit association before the initiation process is correctly triggered. Interactions of ABCE1 with eIF2, eIF3 and eIF5 have been observed in yeast^12^. According to recent structures of initiation complexes, ABCE1 most likely blocks the binding of eIF3B, eIF3G and eIF3I to the small ribosomal subunit by steric hindrances, thus preventing premature assembly of initiation complexes^31^,^40^,^41^,^42^,^43^. Further, a potential interaction of ABCE1 with eIF3B is feasible, based on their positions on the small ribosomal subunit, going along with the known interactions of ABCE1 with the eIF3B, eIF3G and eIF3J subunits of the eIF3 multi-component complex^12^,^31^,^40^,^43^. However, in Archaea, the initiation system is less complex than in Eukarya. Currently, only five archaeal initiation factors are known (aIF1, aIF1A, aIF2/5B, aIF2 and aIF6), showing a different functional spectrum compared with their eukaryotic homologues^44^.

Based on the XL-MS confinement map and supported by the low-resolution cryo-EM reconstruction of the archaeal 30S·ABCE1·ATP PRC, we demonstrated that ABCE1 binds to the GTPase binding center on the small ribosomal subunit, establishing major contacts with S24e and S12. Notably, on ribosomal splitting, the FeS cluster domain undergoes major conformational rearrangements, which position the FeS cluster domain in a cleft between S12 and rRNA (h44) on the small subunit. We thus delineated for the first time the interaction sites and large conformational rearrangements of ABCE1 in the post-splitting/PRC, which forms a potential platform for subsequent translation re-initiation.

## Methods

### Cloning and expression of ABCE1

Full-length ABCE1^wt^ from *S. solfataricus* were cloned with a C-terminal His_6_-tag in pSA4 vector, which is based on a pET15b expression vector^4^,^15^,^45^. For heterologous expression in *Escherichia coli*, the plasmid coding for ABCE1 was co-transformed with the pRARE plasmid (Novagen) coding for rare tRNAs into the BL21(DE3) *E. coli* strain (Novagen). Growth was conducted in lysogeny broth (LB) medium supplemented with 100 μg ml^−1^ ampicillin and 25 μg ml^−1^ chloramphenicol at 37 °C until an OD_600_ (optical density) of 0.6–0.8 was reached and expression was induced by adding 0.35 mM isopropyl-β-D-thiogalactopyranoside. Cells were harvested after 3 h of expression at 30 °C.

### Purification of ABCE1

For protein purification of ABCE1^wt^, all buffers were supplemented with 1 mM of β-mercaptoethanol. Frozen cell pellet was thawed in lysis buffer (20 mM Tris–HCl pH 8.0, 1 mM EDTA, 500 mM NaCl) and disrupted with 4–5 pulses of 3 min on ice using a Branson Sonifier 250 at 70% output. The lysate was centrifuged at 130,000*g* for 30 min. The supernatant was heated for 10 min at 72 °C followed by a second centrifugation at 130,000*g* for 30 min. ABCE1 was purified by immobilized metal affinity chromatography (IMAC, HiTrap Chelating HP, 5 ml, GE Healthcare) using IMAC A buffer (20 mM Tris–HCl pH 8.0, 100 mM NaCl, 20 mM imidazole). After a washing step with 70 mM imidazole (25% IMAC B: 20 mM Tris–HCl pH 8.0, 100 mM NaCl, 200 mM imidazole), ABCE1 was eluted with 200 mM imidazole (100% IMAC B). Fractions containing ABCE1 were pooled and dialyzed against AIEX A buffer (20 mM Tris–HCl pH 8.5) using an Amicon Ultra centrifuge device (30 kDa cut-off, Merck Millipore). The protein was further purified by anion exchange chromatography (AIEX, HiTrap Q column, 1 ml, GE Healthcare) applying a linear gradient from 0 mM to 250 mM NaCl (0–25% of AIEX B buffer: 20 mM Tris–HCl pH 8.5, 1 M NaCl) followed by a final washing step with 1 M NaCl. Protein containing fractions eluted around 15% AIEX B buffer were pooled, dialyzed against HEPES buffer (20 mM HEPES–KOH pH 7.5, 100 mM KCl, 5 mM MgCl_2_), and stored at −20 °C. Protein concentration was determined by ultraviolet absorbance (*ɛ*_280_ 58.720 M^−1^ cm^−1^).

### Purification of ribosomal subunits

To isolate 30S and 50S ribosomal subunits from *S. solfataricus*, a sulfolink resin chromatography was performed as described^46^. Briefly, 5 ml of SulfoLink Coupling Resin (Thermo Scientific) was washed three times with 5 ml coupling buffer (50 mM Tris–HCl pH 8.5, 5 mM EDTA), incubated for 1 h at 20 °C in coupling buffer supplemented with 50 mM L-cysteine and washed again as before. The resin was poured into a spin column device (BioRad, 1,000*g* for 1 min) and equilibrated four times with 5 ml binding buffer (20 mM HEPES–KOH pH 7.5, 5 mM Mg(OAc)_2_, 60 mM NH_4_Cl, 1 mM DTT). *S. solfataricus* cells were resuspended in buffer M (20 mM HEPES–KOH pH 7.5, 5 mM KCl, 10 mM MgCl_2_, 0.5 mM EDTA, 2 mM DTT, 1 mM PMSF, 1 mM Na-heparin, 1 μg RNase-free DNase, 133 U ml^−1^ Ribolock (Fermentas), 1 × protease inhibitor (Serva)), sonicated with two pulses of 1 min on ice using a Branson Sonifier 250 at 70% output, and centrifuged for 30 min at 30,000*g*. The cleared lysate was added onto the SulfoLink column and incubated twice for 15 min on ice. Afterwards, the column was washed three times with binding buffer and elution was performed twice with 1.25 ml of elution buffer (20 mM HEPES–KOH pH 7.5, 10 mM Mg(OAc)_2_, 500 mM NH_4_Cl, 2 mM DTT, 0.5 mg ml^−1^ Na-heparin). The eluate (2.5 ml) was layered onto a 2 ml glycerol cushion (20 mM HEPES–KOH pH 7.5, 10 mM Mg(OAc)_2_, 500 mM KCl, 2 mM DTT, 50% (v/v) glycerol) and centrifuged at 100,000*g* for 15 h at 4 °C to pellet the ribosomes. Pellets were resuspended in 100 μl of cushion buffer without glycerol and incubated for 1 h at 4 °C while shaking. To separate 30S and 50S subunits, 10–30% SDGs (10%/30% (w/v) sucrose, 20 mM HEPES–KOH pH 7.5, 10 mM KCl, 1 mM MgCl_2_) were performed. The resuspended ribosomes were loaded onto the gradients and centrifuged without brake in an SW41 rotor (Beckman Coulter) either for 4 h at 36,000 r.p.m. or for 14 h at 20,000 r.p.m. at 4 °C, respectively. Gradients were fractionated from top to bottom (Piston Gradient Fractionator, Biocomp), recording the absorbance at 254 nm. Fractions containing either 30S or 50S were pooled and concentrated in HEPES buffer using an Amicon Ultra centrifuge device (30 kDa cut-off, Merck Millipore). Concentration of the ribosomes was determined using the absorbance at 254 nm. One OD equals 120 and 60 pmol of 30S or 50S subunit, respectively^47^.

### Purification of 30S·ABCE1·AMP-PNP complex

The 30S·ABCE1·AMP-PNP complex was isolated from SDGs. For this purpose, ABCE1 (10 μM) in HEPES buffer was incubated with 30S (20 OD) and AMP-PNP (2 mM) for 4 min at 73 °C. After cooling on ice (2 min), the samples were loaded on a 10–30% SDG. Fractions containing 30S were pooled and concentrated in HEPES buffer using an Amicon Ultra centrifuge device (30 kDa cut-off, Merck Millipore). Concentration of 30S subunits was determined using the absorbance at 254 nm. One OD equals 120 pmol of 30S. The quality of assembled particles was routinely analysed using negative-stain EM.

### Lysine cross-linking

For lysine-specific cross-linking, 30S·ABCE1·AMP-PNP complexes were formed *in vitro.* Complexes were cross-linked with a heavy-light mixture of disuccinimidyl suberate (DSS-d0/d12, Creative Molecules Inc.), and all measurements done for this study were thereby performed in triplicates. For complex formation, ABCE1 (1 mg ml^−1^) was incubated with a two-fold molar excess of 30S subunit and ADP or AMP-PNP (2 mM each) for 2 min at 73 °C. Either 30- or 80-fold molar excess of DSS cross-linker (2 or 5 mM of DSS) was directly added to this reaction or a further purification step of the PRC via SDG (see above) was performed before adding the cross-linker to obtain a uniform population. The cross-link reaction was incubated for 30 min at 35 °C. To quench the reaction, 0.1 M ammonium bicarbonate was added and incubated for 5 min at 35 °C. Afterwards, the reaction was transferred into acidic conditions by adding 8 M urea and 0.2% (v/v) RapiGest (Waters). Then, 10 mM DTT and 15 mM iodoacetamide were added successively and incubated for 30 min at 37 °C and 600 r.p.m. and for 30 min at 18 °C in the dark, respectively. To digest the cross-linked protein complex, the endoproteinase LysC (1:100, 0.1 μg μl^−1^, Wako) was added and incubated for 4 h at 37 °C and 600 r.p.m. Afterwards, the urea concentration was adjusted to 1.5 M. Trypsin (1:50, 1 μg μl^−1^, Promega) was added and incubated over night at 37 °C. To stop the reaction and allow cleavage of RapiGest 0.5% (v/v), trifluoroacetic acid was added and incubated for 30 min at 37 °C. Subsequently, the peptides were purified and concentrated using C18 micro-spin columns (Harvard apparatus). The columns were equilibrated using 100 μl methanol, 100 μl buffer B (50% acetonitrile, 0.1% formic acid) and two times 100 μl buffer A (5% acetonitrile, 0.1% formic acid) always centrifuged for 1 min at 1,000*g*. The samples were loaded twice with an additional centrifugation step at the end to clean the column. Next, the column was washed four times with 100 μl buffer A and again cleaned with an additional centrifuge step. The elution was performed twice with 75 μl of buffer B. The samples were dried using a Speed-Vac and resuspended in 50 μl of gel filtration buffer (30% acetonitrile, 0.1% trifluoroacetic acid). To analyse the cross-links as well as to separate the cross-linked peptides from others, the samples were examined via gel filtration using a Superdex Peptide PC 3.2/30 column (GE) on a Ettan LC system (GE) at a flow rate of 50 μl min^−1^. Fractions eluting between 0.9 and 1.3 ml were generally pooled, evaporated to dryness and reconstituted in 20–50 μl 5% (v/v) acetonitrile (ACN) in 0.1% formic acid (FA) according to 215 nm absorbance.

### Mass spectrometry

Between 2 and 10% of the collected fractions were analysed by LC–MS/MS using a nanoAcquity UPLC system (Waters Corporation, Manchester, UK) connected online to an LTQ-Orbitrap Velos Pro instrument (Thermo). Peptides were separated on a BEH300 C18 (75 μm × 250 mm, 1.7 μm) nanoAcquity UPLC column (Waters) using a stepwise 60 min gradient between 3 and 85% (v/v) ACN in 0.1% (v/v) FA. Data acquisition was performed using a TOP-20 strategy where survey MS scans (*m*/*z* range 375–1,600) were acquired in the Orbitrap (*R*=30,000) and up to 20 of the most abundant ions per full scan were fragmented by collision-induced dissociation (normalized collision energy=40, activation *Q*=0.250) and analysed in the LTQ Orbitrap. To focus the acquisition on larger cross-linked peptides, charge states 1, 2 and unknown were rejected. Dynamic exclusion was enabled with repeat count=1, exclusion duration=60 s, list size=500 and mass window ±15 p.p.m. Ion target values were 1,000,000 (or 500 ms maximum fill time) for full scans and 10,000 (or 50 ms maximum fill time) for MS/MS scans. All the samples were analysed in at least technical duplicates.

### Identification and analysis of cross-links

Raw files converted to centroid mzXML were searched with xQuest^48^ against sequences of ABCE1 and all the 28 proteins of the small ribosomal subunit from *S. solfataricus* (Supplementary Table 1). Posterior probabilities were calculated with xProphet^30^, and results were filtered with the following parameters: for intra- and mono-links FDR=0.05, min delta score=0.95, MS1 tolerance window±3 p.p.m. and for inter-protein cross-links FDR=0.2, min delta score=0.95, MS1 tolerance window±3 p.p.m. The reliability of the identified inter-protein cross-links was ultimately assessed in the context of available X-ray structures or homology models using Xlink Analyzer (Supplementary Fig. 2a)^37^. For these analyses, an additional conservative cut-off of LD score≥30 was applied within Xlink Analyzer.

### Model building

An *in silico* homology model of the 30S subunit from *S. solfataricus* was constructed to analyse obtained cross-links. To this end, homology models of each known ribosomal protein from *S. solfataricus* (Supplementary Table 1) were constructed using Phyre^2^ (ref. 35). To construct the small 30S subunit of the *S. solfataricus* ribosome, the homology models of the archaeal ribosomal proteins were aligned to the known small ribosomal subunit from *S. cerevisiae* (pdb: 3U5G, 3U5F)^36^. Yeast ribosomal proteins are thereby named according to the new nomenclature of ribosomal proteins, while the archaeal r-proteins hold their UniProt entry name going along with the MS analysis^19^. A model of ABCE1 in the closed state is positioned according to the cryo-EM map of the pre-recycling complex (pdb: 3J16)^8^. Finally, the XlinkAnalyzer tool for Chimera was used to analyse and certify the obtained cross-links^37^.

### Sample preparation for Cryo-EM

A concentration of 50 nM *S. solfataricus* 30S was incubated with 100 nM *S. solfataricus* ABCE1^E238A/E485A^ and 2 mM of AMP-PNP in binding buffer (20 mM Tris pH 7.5, 100 mM KCl, 5 mM MgCl_2_, 2 mM DTT) for 5 min at 25 °C. Samples were vitrified on carbon supported grids by standard procedure for cryo-EM imaging.

### Electron microscopy and image processing

Freshly prepared sample was applied to 2 nm pre-coated Quantifoil R3/3 holey carbon supported grids and vitrified using a Vitrobot Mark IV (FEI Company) and visualized on a Spirit TEM (FEI Company) with about 20e^−^ Å^−2^ at a nominal magnification of × 105,000 with a nominal defocus between −1 μm and −3.5 μm. Automatic particle detection was performed by the programme SIGNATURE^49^. Initial *in silico* sorting of the data set consisting of 54,800 particles in total was performed using the SPIDER software package^49^. Classes were obtained by competitive projection matching in SPIDER^50^,^51^. The final 30S·ABCE1 data set contained 19,500 particles and the final resolution was 17 Å (Fourier shell correlation 0.5).

For interpretation of the 30S·ABCE1 electron density at a molecular level, the models for the *Pyrococcus furiosus* 30S subunit (4V6U)^52^ and ribosome-bound ABCE1 in (3J15)^8^ were fitted as rigid bodies using UCSF Chimera. The FeS cluster domain was repositioned by a rotation of ∼160° around a hinge (residues 76–78) into an unaccounted electron density near ribosomal protein S12. This repositioning results in a close contact between lysine 60 of ABCE1 (Lys64 in *P. furiosus*) and lysine 40 of S12 and is consistent with above described XL-MS data.

### Data availability

The structural coordinates of ABCE1 and the electron density map of the archaeal PRC 30S·ABCE·ATP-PNP have been deposited in the Protein Database under ID code 5LW7 and the electron microscopy databank under code EMD-4113. The data that support the findings of this study are available from the corresponding author on reasonable request.

## Additional information

**How to cite this article:** Kiosze-Becker, K. *et al*. Structure of the ribosome post-recycling complex probed by chemical cross-linking and mass spectrometry. *Nat. Commun.* **7,** 13248 doi: 10.1038/ncomms13248 (2016).

**Publisher's note:** Springer Nature remains neutral with regard to jurisdictional claims in published maps and institutional affiliations.

## Supplementary Material

Supplementary Information Supplementary Figures 1 - 5; Supplementary Tables 1 - 3

Supplementary Data 1 Data XQuest

## Figures and Tables

**Table 1:** Identified inter cross-links between ABCE1 and ribosomal proteins.

**ABCE1**		**Ribosomal proteins**		**Identified inter cross-links**		
**Domain**	**Residue**	**Name**	**Residue**	**SDG-purified PRC**	**PRC with AMP-PNP**	**PRC with ADP**
NBD1	136	S24e	119	+	+	−
NBD1	136	S24e	113	+	+	−
NBD1	133	S24e	119	+	−	−
NBD1	192	S24e	119	+	−	−
NBD1	141	S24e	119	+	−	−
NBD1	153	S24e	113	+	+	−
NBD1	141	S24e	113	+	−	−
FeS	60	S12	40	+	−	−
