# Supplementary material for: Structure of the ribosome post-recycling complex probed by chemical cross-linking and mass spectrometry
Source: Nat Commun. 2016 Nov 8;7:13248. doi: 10.1038/ncomms13248 (PMC5105147; doi:10.1038/ncomms13248)
Supplement: Supplementary Information — Supplementary Figures 1 - 5; Supplementary Tables 1 - 3 [file ncomms13248-s1.pdf]

Supplementary Figures

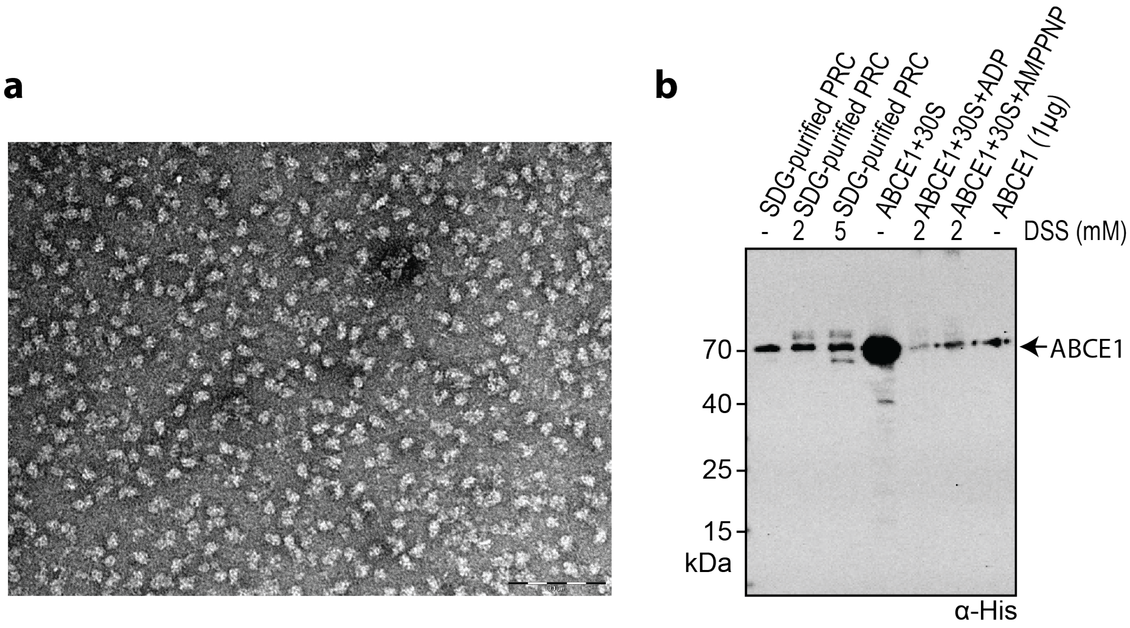

Supplementary Figure 1: Quality control of the purified post-recycling complex.

a, The quality of purified PRCs was analyzed via negative stain EM, depicting an even distribution in particle size and a clear homogeneity of the sample (bar 100 nm). **b,** Immunoblotting of the PRC samples before and after cross-linking (with 30- or 80-fold molar excess of DSS) displays a distinct band for ABCE1 and no undesired aggregation upon cross-linking.

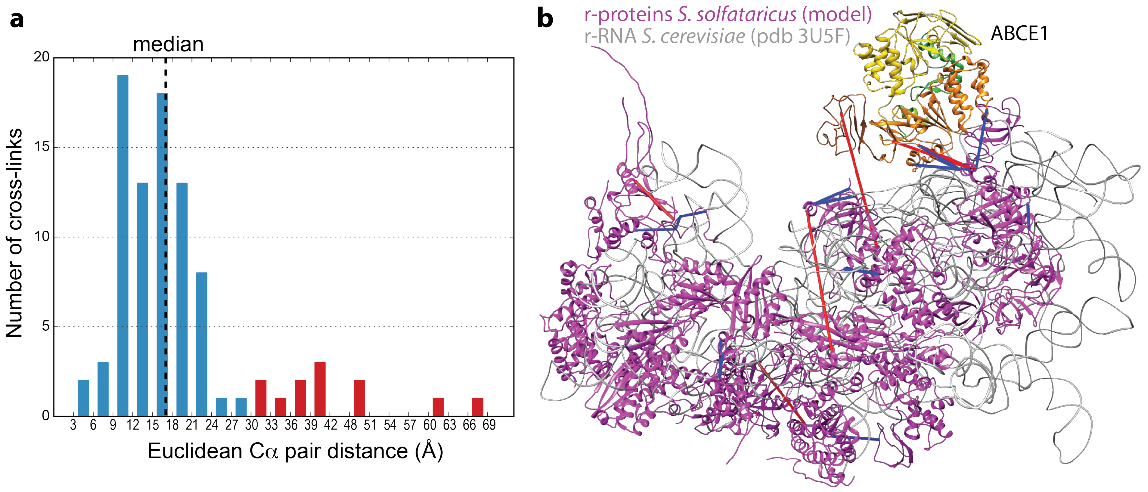

Supplementary Figure 2: Inter cross-links.

a, A histogram of all identified distance restraints of the post-recycling complexes with the median distance at 17 Å and with 86.7% of all cross-links below 30 Å. **b,** All identified inter cross-links are depicted using an *in silico* constructed model of the *S. solfataricus* 30S, which was built by aligning the homology models of the archaeal ribosomal proteins (magenta) to the small ribosomal subunit from *S. cerevisiae* (pdb: 3U5G/F, rRNA: gray) and positioning ABCE1 according to the cryo-EM map of the rescue/pre-recycling complex (pdb: 3J16). Inter cross-links between different r-proteins as well as between ABCE1 and r-proteins are depicted as blue and red lines (cross-links ≤ 30 Å blue and > 30 Å red).

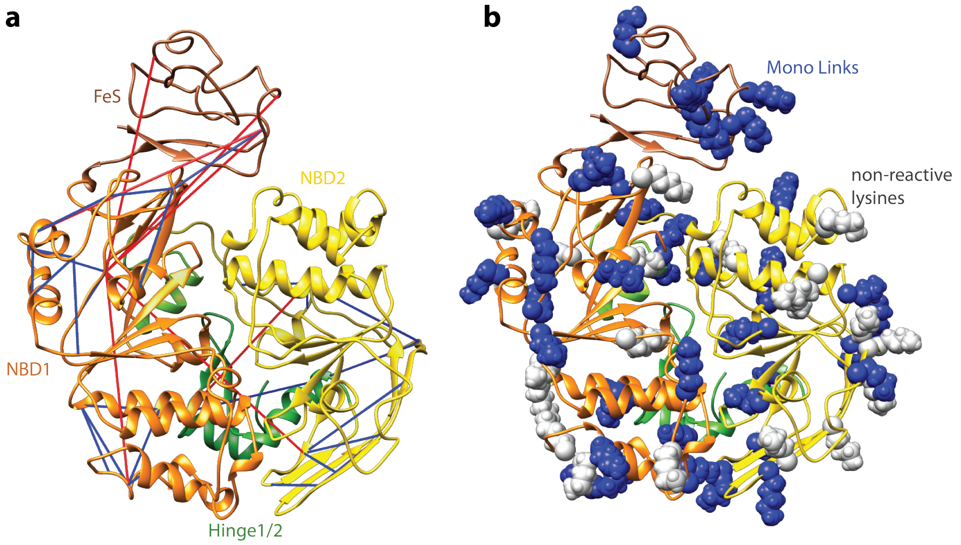

Supplementary Figure 3: Intra-cross links and mono links of ABCE1.

a, Intra ABCE1 cross-links combined from all independent experiments are certified using a model of the closed state of ABCE1 and are depicted as blue and red lines (cross-links ≤ 30 Å, blue and > 30 Å red). **b,** Lysine residues, which formed a mono-link by reacting with the DSS molecule, are depicted as blue spheres, while non-reactive lysines are shown in grey.

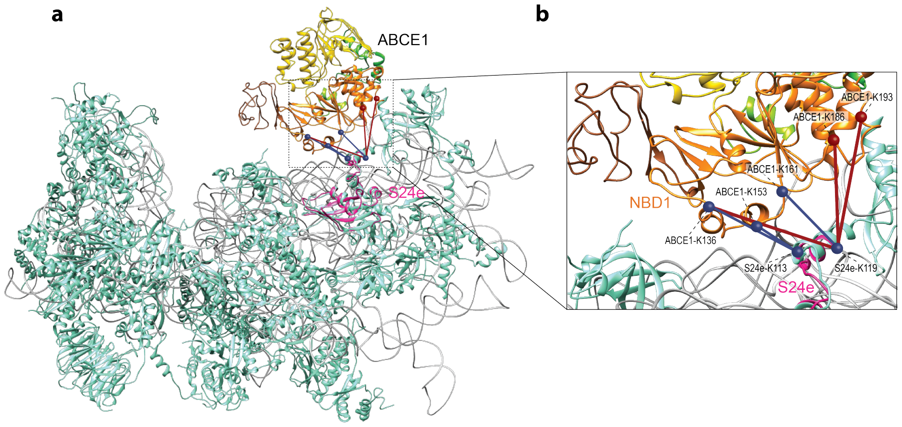

Supplementary Figure 4: Inter cross-links of the PRC AMP-PNP sample.

a, Orientation of ABCE1 in the PRC, based on the identified inter cross-links with the archaeal ribosomal protein S24e (purple, **b**), depicted as blue and red lines. Blue lines indicate cross-links with a length below 30 Å and red lines cross-links longer than 30 Å. Identified inter cross-links were certified with a constructed *in silico* model of the *S. solfataricus* 30S by aligning the homology models of the archaeal ribosomal proteins to the small ribosomal subunit from *S. cerevisiae* (pdb: 3U5G/F, r-proteins: cyan, rRNA: gray) and positioning ABCE1 according to the cryo-EM map of the rescue/pre-recycling complex (pdb: 3J16).

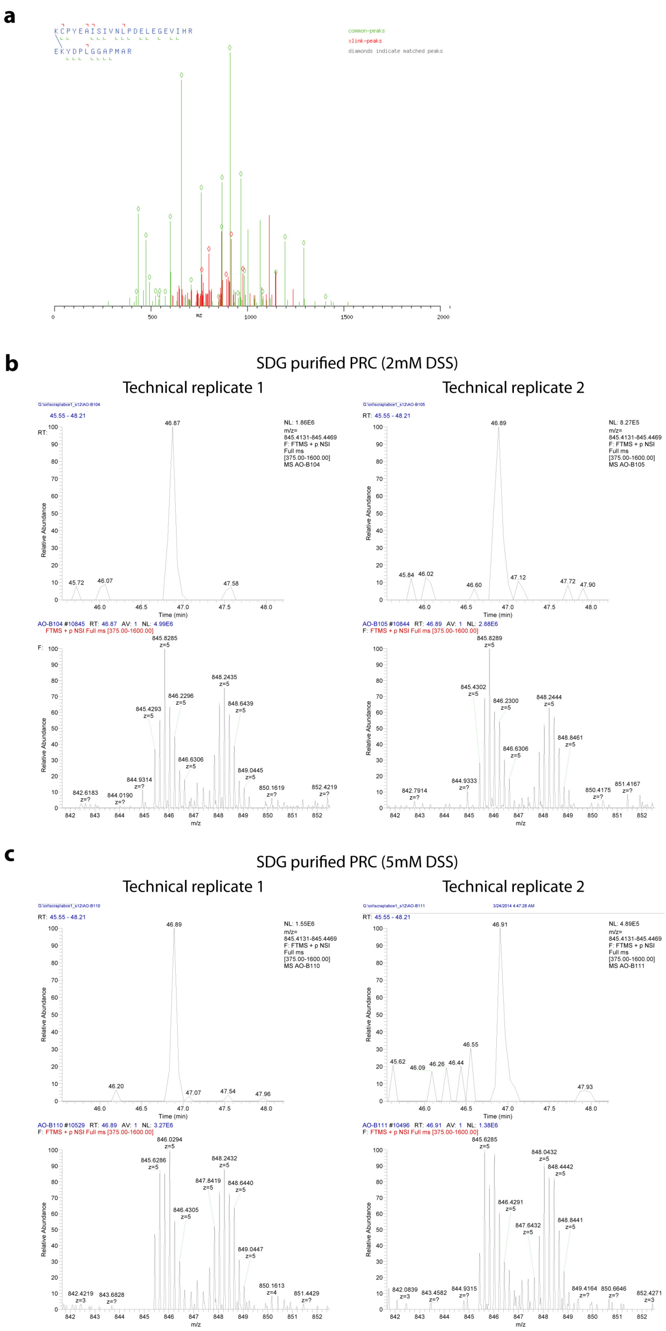

Supplementary Figure 5: Spectra of the S12-ABCE1 cross-link.

a, Fragment ion spectrum of two cross-linked peptides from ABCE1 (KCPYEAISIVNLPDELEGEVIHR) and RS12 (EKYDPLGGAPMAR). Ion series for both peptides are indicated, red peaks in the spectrum correspond to fragment ions containing the cross-linked lysine pair (cross-link peaks), green peaks correspond to fragment ions that do not include the linked site (common peaks). All fragment ions that match a peak in the spectrum are marked with diamonds. The ABCE1-RS12 cross-link was consistently present in two independent cross-linking reactions: one was carried out using 30-fold molar excess of DSS (2 mM DSS) **(b)** and the second using 80-fold molar excess of DSS (5 mM DSS) **(c)**. In both cases, the characteristic precursor ion pattern corresponding to the peptides cross-linked with the light and heavy version of DSS is apparent in the MS1 spectra of both technical replicates (repeated injection of the same sample). For each technical replicate, the upper panel shows the extracted ion chromatogram for the light version of the cross-linked peptide ion that is consistently eluting at a retention time of ~46.9 min in all samples. The bottom panel shows the corresponding MS1 spectrum derived from the maximum intensity point of the extracted ion chromatogram.

Supplementary Tables

Supplementary Table 1 Protein database used for peptide identification

UniProt entry	UniProt name	Gene name	Sequence	Length (aa)
Q980K5	Q980K5	SSO0287/ABCE1/RLI1	MRVAVINYDFCKPDKCNLECINFCPVDRSGGKAI<br>ELSEIVKGKPVIYEETCIGCGICVKKCPYEAISI<br>VNLPDELEGEVIHRYKVNGFKLFGLPTPKNNTIL<br>GVLGKNGVGKTTVLKILAGEIIPNFGDPNSKVGK<br>DEVLKRFRGKEIYNYFKELYSNELKIVHKIQYVE<br>YASKFLKGTVNEILTKIDERGKKDEVKELLNMTN<br>LWNKDANILSGGGLQRLLVAASLLREADVYIFDE<br>PSSYLDVRERMNMAKAIRELLKNKYVIVVDHDLI<br>VLDYLTDLIHIIYGESSVYGRVSKSYAARVGINN<br>FLKGYLPAENMKIRPDEIKFMLKEVSDLDLSKDL<br>KTKMKWTKIIKKLGDFQLVVDNGEAKEGEIIGIL<br>GPNGIGKTTFARILVGEITADEGSVTPEKQILSY<br>KPQRIFPNYDGTVQQYLENASKDALSTSSWFFEE<br>VTKRLNLHRLLESNVNDLSGGELQKLYIAATLAK<br>EADLYVLDEPSSYLDVEERYIVAKAIKRVTRERK<br>AVTFIIDHDLSIHDYIADRIIVFKGEPEKAGLAT<br>SPVTLKTGMNEFLRELEVTFRRDAETGRPRVNKI<br>GSYLDRVQKERGDYYSMVLSTQ	600
P95993	RS2	rps2	MKVTNLSEKEERGGELTEAEKEELRKSEKGAIIE<br>LLVPVDTYLSAGVHIGTHSCTKYMESFVYRVRAE<br>GLYVLDVRKIDERLRIAAKFLSRYDPQDIIVVAS<br>RPYAYRPVQKFAEVVGSRALVGRIIPGTFTNPYL<br>STYIEPKVLLVSDPRTDTQAIKEAAKVGIPIVAF<br>ADTDAKIDYIDLIIPANNKGRKSLALLYWALARQ<br>ILRERRVIPPDGDLAVPVSEFEMRLVQ	231
Q9UXA0	RS3	rps3	MPNIKRYFLEKSIVKVKIDEYLAKQYYNAEYAGV<br>EVLKTPIGTRVIIYAGRPSMIIGRGGRNIKQLAQ<br>IFEKVFGLENPQITITNVENPELNARVMAFRLAI<br>ALEKGYHFRRAAFISMRRIMNAGALGAEIIISGK<br>LTTERARYEKLKEGIVYKSGQQLEKMIDRAIAIA<br>MLKPGIFGVEVVITKPLKIEDKINLKESPSVPQE<br>VSVTNVTFIEESSQKSEEKSEGEKE	229
P95987	RS4	rps4	MGDPKKSRKKWETPGHPWIKERIGYEQELLGKYG<br>LRNKREIWIAQSIIRKFRHQARSLLALPPAERAV<br>REKQLVGKLLKMGLLKKETATVDDILSLTEQDLL<br>ERRLQTIVYKKGLSNTIYQARQLITHGHIAVNGK<br>RVTSPGYIVNVDEENLIDYYVTSSFKSRPPVMSQ<br>QEGGEIGVKQA	181
Q9UX87	RS5	rps5	MAEEVPSLNIEEWKPRTSIGSLVKEGKISSIKEL<br>FDRNLPITEPEIVDVLLPKLKYEVVDIKVVQKQT<br>DAGEISRYKVLVIMGNMDGYVSIGTGKAKQLRVA<br>IQKAIRDAKMNIIPVRRGCGSWQCTCGEPHSLPF<br>KVVGKAGSVEVDLLPAPKGTGLVVGSVLKTLLTY<br>AGIKDAWSTTKGETRTTENFVRAGYSALYNTYKF<br>VTLQDWVRKR	214
P35026	RS7	rps7	MSLENLQLDIKVFGKWDTKVEIRDPSLKKYISLM<br>PVYLPHTGGRHEHRRFGKAKVPIVERLINQIMRP	193

			GRNKGKKHLAYNIVKLAFDIIYLKTGQNPIQVLV RAIENSAPREEVTRIMYGGIVYYVAVDVSPQRRI DLALRHIATGAKDASFNNPKPIEEVLAEEIIAAA NNDSKSFAIKRKEEIERIALSSR	
Q9UX92	RS8	rps8	MVFVNPLANALTSIYNNEMRRNKQAIIMPASKLV INVLRVMQKEGYVGEFEYIDDGRWGKITVQLLGR VNKCGPITPRYPLSYRQMIALPDYVRRYLPSKEI GIIIVSTSKGVMSHKEAARLRIGGVALGYVY	133
P95992	RS9	rps9	MSEEQKLVISSARRKTARATCYIYAGKGRVFVNN VPIELIPIEMVRLKIMEPLLLAGNDIRSKIDAKI ITYGGGIMGQADAARMALARALVKFTGSKELEKI YRAYDRTMLAGDPRQTESEKWMRYSARRWRQKSY R	137
P35027	RS10	rps10	MPTKARIRLWSTNVENLNYVITQIRGIVEKTGIE MRGPIPLPTSKLEVPIMRLPHGEGRKKWEKWEMR VHKRLIDIAADERVMRQLMRVRVPEDVYIEIQLI	102
P95988	RS11	rps11	MSSRREIRWGIAHIYASQNNTLLTISDLTGAEII SRASGGMVVKADREKSSPYAAMLAANKAASDALE KGIMALHIKVRAPGGYGSKTPGPGAQPAIRALAR AGFIIGRIEDVTPIPHDTIRRPGGRRGRRV	132
P39573	RS12	rps12	MVKSKSPKGIYAARKLRLKRLKFRRSQRKYKTKI LKLKEKYDPLGGAPMARGIVLEKVGIESRQPNSA VRKCVRVQLVRNGRVVTAFVPGDGGVNFIDEHDE VIITGIGGTLGRSMGDLPGVRYKVIMVNGVSLDA LYKGKKQKPVR	147
P95986	RS13	rps13	MSQQFKYVVRIFGQDVDGTMKLPYALAMVKGIGY NTAKAIIRKLGMDPNARLGELSDAEVKKVESVIS DHTIKGLPSWLYNRRKDYESGLDLHLVTSDLIFY VRNDIEREKKSRSWRGVRHSLGLKVRGQRTRTTG RTGMTIGVARKKAAQPQSQQSSSQQQKSS	136
Q97ZQ5	RS14Z	rps14	MGKYKPPAERKYGKGVQSCQRCGSKDSVIQKYGI YLCRQCFREVAYELGFRKYW	54
Q980A8	RS15	rps15	MNKRRAKGKSHSIRPARAGAPKWVRLTREEVEML VEELAKRGYTPSMIGIILRDQYGIPLVKQIVGKK VTQILEERGLAPQIPEDLFNLIRKAVNVRRHINE YPRDKTAKKGLEEIESKIRRLTRYYKGIGKLPQE WVYDPAKAELLVAGAS	152
Q9UX98	RS17	rps17p	MVSKGKTVKDPGIPNITIPEKVCEDEDCPYHGSL RVRGITLEGVIVKYRGTKAAVIERQYLYYDSKYK RYERRRSRIHAHVPPCINVREGDKVIIGECRPLS KSISFVVLGKVS	114
D0KTC1	D0KTC1	rps19p	MSLEIPPEWKNFKYRGKSIDELLNMPMDEFIKLL PSRQRRSLKRGFTDAQRHLLEKVRKYRREGKFNK TIKTHVRNLVILPELIGLKMAVYNGKEFVEFTVT PEMIGHYLGEYSITTKKVEHGEPGLKATRSSLFL AMKG	119
Q9UXD4	RS3A	rps3ae	MSAKGGAIKDKWKMKKWYSVITPKAFGEVSLGST PAYDITQTIGRRVETTLYDLTGDFSQVYVHLYFK IIGNEGDRLITRFVGHELSRDYLRSLIRRKSSKI NSIFDVTTKDGYVVRVKGLVLTTYKCHQSQKTAI RKIINETVSKKASELSFDDFTQEVVFGRLANEIF EAAKKIYPLRKAEIEKTKVLKVPENLGKQVESSS VSSG	208

Q9UX94	RS4E	rps4e	MAHITRFEAPWFLMISKKQYKWTVRPNAGPHSIE<br>KSIPLAVVIRDYLKLAGTIREAKHIIFDGKVLVD<br>GKVRKDYKYPVGLMDIVSIPSADLYFRVLPDNVR<br>FMRFSKISADEARYKYVRIINKTTIKEGRIQLNL<br>EDGRNILVDKETAKNFKTLMTLKIELPSQQILDS<br>FTISERSYAIFVGGRNVGIHGIVKNINLSKFKSR<br>KYSVITLESRDGNTYQTNIMNVMSIGREKSDLRV<br>D	239
Q980A6	RS6E	rps6e	MPDFKIVISDPQSVEPKRIKVKVKASDQVKSITG<br>EKDGKAVPQAKVNEKTKQLLNVDTLLTLEITKQE<br>GDKKVKVKGHFKVDVDNSVPDNEVWISKTMAEKF<br>GAEDFEAFAYRTKTLQISVDQNKATNLVGLKIGD<br>VFEANQLIGLPVKLKITGGSDNSGFPMRFDVIGA<br>AKRKILLSGPPGFYPNENGERRRKTIRGNTISQE<br>IVQINTIIVR	214
Q980W3	RS8E	rps8e	MGFYQGPDNRKITGGLKGKHRDKRKYEIGNPPTF<br>TTLSAEDIRIKDRTLGGNFKVRLKYTTTANVLDP<br>ATNTAKKVKILEILETPANKELARRGIIIRGAKI<br>RTEAGLAVVTSRPGQDGVINAVLLKNESQRS	133
Q980K7	RS17E	rps17e	MGNIYTKDIKRIVKEIYDRYKDEIKDDYNTNKQI<br>VIRYVDVKSKKVRNRIAGYLTRYYKIMKEKETSP<br>AEEKEEISEEI	79
Q980F7	Q980F7	rps19e	MSLIMITAEMVPPDLLIKRLAIYLKENVKTVDPP<br>EWALLAKTASFKERVPDNAEDWWYIRAASLLRKL<br>YVNSIIGIEKTRTIYGGRKRRGTRPEKFVKAPGH<br>VNRLIFQQLEKAGLVQKIKNKGRSLSPKGRSLLD<br>KLALEIFKELAENNTSLKVYLE	158
Q97ZY6	RS24	rps24e	MESQAKVKISDKAEGIIERDMQNSVIGRREISLK<br>VYHMGSGTPSRKDIIKAIIQALGSQENLVVVRKI<br>STSYGAGISNVKLHIYKSREILEKVEPKYLLDRD<br>AGTKQKKGGSKGGQGAKG	120
Q97ZZ6	RS25	rps25e	MGGASKKPISTMEKRLKKEAEKQQKAEEKKKGPS<br>KTGKEIISRAVTIDEETKKKVLDEIKKESIITPY<br>ALATKSGISISVARKILKELENQNVVKLYSKNRR<br>LEIYIAAS	110
Q97ZR1	Q97ZR1	rps26e	MPKKRENRGRRKGDKGHVGYISCDQCGARVPEDK<br>AVCVTKMYSPVDASLASELEKKGAIIARYPVTKC<br>YCVNCAVFLGIIKIRAENERKQKARLR	95
Q97Z80	RS27	rps27e	MMRKLRVLIPEPKSRFLRVKCPNCGNEQTIFSHA<br>TFPVRCLSCGTELVYSMGGKAKIVGEVVRIMG	66
Q97ZY7	RS27A	rps27ae	MLELNKRKEEAKVAKEQKVKAIVRTYYVIEGNKV<br>KLKNKKCPRCGSIMAHHLKPNERWSCGKCGYTEF<br>IGASKKR	75
Q980Q5	RS28	rps28e	MSEKTQQSQGSSIIEEFGFPAEVIQILDRTGVTG<br>EVTQVRVRVLEGRDKGRILTRNVKGPVRVGDILI<br>LRETEREARKITTKR	83
Q97ZH4	Q97ZH4	rps30E	MPSHGSLTKAGKVRSQTPKIQPKEKHKEVPRVRN<br>RKEYEKRVVKARQQAPAR	52

Supplementary Table 2 Inter cross-links between ABCE1 and ribosomal proteins (ld-score cutoff ≥ 30)

ABCE1		r-proteins					Independent cross-linked peptides						
							PRC preparation 2				PRC preparation 1		
peptide	*residue*		*peptide*	*residue*	*modeled to 40S yeast (pdb:3U5G/F)*	*distance [Å]*	*SDG purified (5mM DSS)*	*SDG purified (2mM DSS)*	*ADP*	*AMP-PNP*	*SDG purified (2mM DSS)*	*ADP*	*AMP-PNP*
VGKDEVLKR	136	S24e	GGQGAKG	119	eS24	31.3	1	1	-	-	2	-	2
VGKDEVLKR	136	S24e	GGSKGGQGAK	113	eS24	22.4	1	3	-	2	3	-	-
ILAGEIIPNFGDPNSKVGKDEVLK	133	S24e	GGQGAKG	119	eS24	40.6	-	1	-	-	-	-	-
GKKDEVK	192	S24e	GGQGAKG	119	eS24	26.9	-	1	-	-	-	-	-
VGKDEVLKR	141	S24e	GGQGAKG	119	eS24	28.4	-	1	-	-	-	-	-
EIYNYFKELYSNELK	153	S24e	KGGSKGGQGAK	113	eS24	11.6	1	-	-	1	-	-	-
VGKDEVLKR	141	S24e	KGGSKGGQGAK	113	eS24	20.0	1	-	-	-	-	-	-
KCPYEAISIVNLPDELEGEVIHR	60	RS12	EKYDPLGGAPMAR	40	uS12	59.5*	1#	1	-	-	-	-	-

r-protein 1			r-protein 2										
peptide	*residue*	*r-protein*	*peptide*	*residue*	*r-protein*								
SGQQLEKMIDR	161	RS3	YVDVKSK	42	RS17E	15.2	1	1	1	1	1	1	1
KAAQPQSQQSSSQQQK	148	RS13	KVEHGEPGLK	119	RS19	15.8	2	1	-	-	1	-	-
KAAQPQSQQSSSQQQK	148	RS13	KVEHGEPGLKATR	128	RS19	16.3	1	1	-	1	-	-	-
HIIFDGKVLVDGK	64	RS4E	LHIYKSR	85	RS24	17.6		1	1	1	-	-	-
NDIEREKK	111	RS13	KVEHGEPGLK	119	RS19	16.5	-	1	-	1	-	1	1
SQTPKIQPK	19	RS30E	KQKPVR	142	RS12	16.2		1	-	-	-	1	1
IGYEQELLGKYGLR	32	RS4	RVVKAR	44	RS30E	13.9	-	1	-	-	-	-	-
AKIVGEVVR	56	RS27	KIYPLR	175	RS3A	20.8	1	-	-	-	1	1	1
AIRDAKMNIIPVR	111	RS5	MPSHGSLTKAGK	9	RS30E	63.9	-	-	-	-	-	-	1
SQTPKIQPK	19	RS30E	KQKPVR	144	RS12	18.0	-	-	-	-	-	-	1
KAEIEK	181	RS3A	KITTKR	82	RS28	33.8	-	-	-	-	-	-	1
IEDKINLK	192	RS3	YVDVKSK	42	RS17E	21.5	-	-	-	-	1	-	1

*Conformational reorganization of the FeS-cluster domain from 59.5 to 17.5 Å before and after ribosome splitting; #this cross-link was identified with high confidence in the SDG purified (2mM DSS) sample and the characteristic precursor ion pattern corresponding to the peptides cross-linked with the light and heavy version of DSS was apparent in the MS1 spectra of both technical replicates also for the SDG purified (5mM DSS) sample, see Supplementary Fig. 5 for details.

Supplementary Table 3 Intra cross-links within ABCE1

						Independent cross-linked peptides						
						PRC preparation 2				PRC preparation 1		
peptide	***res.1***	***peptide***	***res.2***	***dist. [Å]***	***domain***	***SDG purified (5mM DSS)***	***SDG purified (2mM DSS)***	***ADP***	***AMP-PNP***	***SDG purified (2mM DSS)***	***ADP***	***AMP-PNP***
IRPDEIKFMLK	325	YIVAKAIK	500	9.4	NBD2/NBD2	1	2	1	1	-	-	-
GEPEKAGLATSPVTLK	539	KLGDFQLVVDNGEAK	352	10.5	NBD2/NBD2	1	1	1	1	1	1	1
IIVFKGEPEK	534	WTKIIK	348	15.9	NBD2/NBD2	3	3	1	3	1	-	-
MNMAKAIR	253	ELLKNK	260	12.1	NBD1/NBD1	2	2	-	1	1	-	1
VNKIGSYLDR	577	ELLKNK	260	14.1	Hinge2/NBD1	1	1	1	1	-	1	1
GKEIYNYFK	146	VSKSYAAR	296	12.5	NBD1/NBD1	2	1	2	2	-	1	1
AGLATSPVTLKTGMNEFLR	550	EVSDLDLSKDLK	338	17	Hinge2/NBD2	1	1	-	1	-	-	-
EIYNYFKELYSNELK	153	VGKDEVLKR	141	9.6	NBD1/NBD1	-	1	1	1	-	-	-
VNKIGSYLDR	577	MNMAKAIR	253	8.2	Hinge2/NBD1	1	1	-	1	1	-	-
KLGDFQLVVDNGEAK	352	WTKIIK	348	13.4	NBD2/NBD2	1	1	1	1	1	1	1
LFGLPTPKNNTILGVLGK	97	VSKSYAAR	296	13.6	NBD1/NBD1	1	1	1	1	-	1	1
IIVFKGEPEK	534	WTKIIKK	351	16	NBD2/NBD2	1	1	-	-	-	-	-
TTVLKILAGEIIPNFGDPNSK	117	YKVNGFK	84	17.2	NBD1/NBD1	1	1	1	1	-	-	-
YIVAKAIK	500	EVSDLDLSKDLK	338	20.6	NBD2/NBD2	1	1	-	-	1	-	-
EIYNYFKELYSNELK	153	VGKDEVLK	136	8	NBD1/NBD1	1	1	-	2	-	-	1
ILAGEIIPNFGDPNSKVGK	133	YKVNGFK	84	16.4	NBD1/NBD1	2	2	-	1	1	2	1
ILAGEIIPNFGDPNSKVGK	133	DEVLKR	141	10.8	NBD1/NBD1	1	1	1	1	-	-	1
FMLKEVSDLDLSK	329	YIVAKAIK	500	11.8	NBD2/NBD2	1	1	-	-	-	-	-
GTVNEILTKIDER	186	GKKDEVK	193	10.2	NBD1/NBD1	1	1	1	1	-	-	-
GKEIYNYFK	146	VGKDEVLKR	141	9.9	NBD1/NBD1	1	1	2	2	-	1	-
ELYSNELKIVHK	161	VGKDEVLK	136	14.5	NBD1/NBD1	2	2	1	1	-	-	1
GKKDEVK	192	ELLKNK	260	15	NBD1/NBD1	1	-	1	1	-	-	1
MNMAKAIR	253	GKKDEVK	193	17.7	NBD1/NBD1	1	-	-	-	-	-	-
IIVFKGEPEK	534	MKWTKIIK	345	21.5	NBD2/NBD2	-	-	-	1	-	-	-
VNKIGSYLDR	577	YIVAKAIK	500	27.7	Hinge2/NBD2	-	-	1	1	-	-	-
VAVINYDFCKPDK	12	YKVNGFK	84	19.1	FeS/NBD1	-	-	-	-	-	1	1
VSKSYAAR	296	GKKDEVK	193	37.6	NBD1/NBD1	-	-	-	-	-	1	1

VNKIGSYLDR	577	AGLATSPVTLKTGMNEFLR	550	19.8	Hinge2/NBD2	-	-	1	1	-	-	1
VAVINYDFCKPDKCNLECINFCPVDR	12	VSKSYAAR	296	39.2	FeS/NBD1	-	-	1	-	-	1	1
DEVLKR	141	ILAGEIIPNFGDPNSKVGK	136	8.4	NBD1/NBD1	-	-	-	-	1	-	-
VAVINYDFCKPDKCNLECINFCPVDR	15	VSKSYAAR	296	43.9	FeS/NBD1	-	-	-	-	-	-	1
SGGKAIELSEIVK	32	VSKSYAAR	296	37.0	FeS/NBD1	-	-	-	-	-	-	1
IIVFKGEPEK	534	VSKSYAAR	296	41.8	NBD2/NBD1	-	-	-	-	-	-	1
